# A reproducible epithelial transcriptional state combines interferon-associated and secretory programs in pancreatic ductal adenocarcinoma

**DOI:** 10.3389/fgene.2026.1907806

**Published:** 2026-08-12

**Authors:** Xingyu Mi, Shifan Wu, Liang Xiao

**Affiliations:** 1 Department of Liver Surgery, Xiangya Hospital, Central South University, Changsha, China; 2 Department of Hepatobiliary Surgery, Hunan Provincial People’s Hospital, Changsha, China

**Keywords:** epithelial plasticity, interferon signaling, pancreatic ductal adenocarcinoma, secretory epithelial program, single-cell RNA sequencing

## Abstract

Pancreatic ductal adenocarcinoma (PDAC) exhibits extensive epithelial plasticity, yet epithelial states associated with interferon signaling remain incompletely defined. We analyzed cleaned epithelial cells classified as singlets in two independent PDAC single-cell RNA-sequencing cohorts, GSE155698 and GSE212966, and constructed a composite score combining an interferon-associated module (IFNcore) with an epithelial-secretory module (Secretorycore). VM-high cells consistently co-expressed representative genes from both programs. Within individual PDAC tumors, IFNcore and Secretorycore were positively associated in 14 of 17 GSE155698 samples and 5 of 6 GSE212966 samples. Sample-level effect sizes and leave-one-gene-out analyses further identified IRF1, STAT1, and ELF3 as candidate factors associated with distinct components of the state. The principal findings were preserved after doublet filtering and remained detectable in Harmony- and CopyKAT-based robustness analyses. In the independent GSE62452 cohort, the composite score correlated with a non-overlapping interferon signature among 69 PDAC tumors (Spearman rho = 0.584, P = 1.42 × 10^-7). In TCGA-PAAD, a higher continuous score was associated with poorer overall survival in univariate Cox analysis, although the association was attenuated after adjustment for age, sex, stage, and grade. These findings define a reproducible PDAC epithelial transcriptional state in which interferon-associated activity coexists with secretory epithelial identity and nominate IRF1, STAT1, and ELF3 for subsequent functional investigation.

## Introduction

Pancreatic ductal adenocarcinoma (PDAC) is a highly lethal malignancy characterized by extensive molecular, cellular, and microenvironmental heterogeneity (Siegel et al., 2024; Kamisawa et al., 2016). Bulk transcriptomic studies have identified classical/progenitor, basal-like/squamous, and related epithelial phenotypes (Collisson et al., 2011; Moffitt et al., 2015; Bailey et al., 2016; Puleo et al., 2018; Chan-Seng-Yue et al., 2020). More recent single-cell and spatial studies have shown that these categories are not fixed entities but are accompanied by continuous epithelial-state variation shaped by tumor evolution, therapy, and local microenvironmental context (Peng et al., 2019; Lin et al., 2020; Elyada et al., 2019; Wang et al., 2021; Cui Zhou et al., 2022; Werba et al., 2023; Oh et al., 2023; Loveless et al., 2025).

This expanded view of PDAC heterogeneity has also highlighted an interpretive challenge. Inflammatory and stress-related transcriptional programs may reflect stable tumor-cell adaptations, transient responses, or technical and cellular admixture. Establishing whether such programs recur within epithelial compartments across independent datasets therefore requires both cell-level resolution and evidence of consistency across biological samples.

Interferon signaling has been implicated in PDAC progression, immune regulation, and treatment response (Muthalagu et al., 2020; Ischenko et al., 2021; Vonderhaar et al., 2021; Perevalova et al., 2024; Alsamman and El-Masry, 2018). At the same time, ductal secretory genes, including S100P, PIGR, TFF1, AGR2, CLDN3, and MUC13, are recurrent features of PDAC epithelial identity and plasticity (Subbalakshmi et al., 2023; Luk et al., 2018; Dumartin et al., 2011; Chauhan et al., 2012; Deng et al., 2008; Borka et al., 2007). Whether interferon-associated activity reproducibly coexists with this secretory epithelial program within the same tumor-associated epithelial state has not been systematically resolved.

We hypothesized that interferon-associated and epithelial-secretory programs define a reproducible transcriptional axis within PDAC epithelial cells and that distinct transcriptional factors preferentially associate with its component programs. We tested this hypothesis in two independent single-cell cohorts, evaluated consistency across individual tumors, performed complementary robustness analyses, and examined the resulting signature in GSE62452 and TCGA-PAAD. The study was designed to define a reproducible epithelial state and prioritize biologically interpretable candidate factors rather than to establish a causal regulatory mechanism.

## Methods

### Data sources and single-cell preprocessing

Single-cell RNA-sequencing data from the publicly available GSE155698 and GSE212966 pancreatic ductal adenocarcinoma (PDAC) cohorts were obtained from the Gene Expression Omnibus (Barrett et al., 2013). Bulk transcriptomic data from GSE62452 and RNA-sequencing and clinical data from TCGA-PAAD were used for external and clinical validation (Barrett et al., 2013; Cancer Genome Atlas Research NetworkCancer Genome Atlas Research Network, 2017). All datasets were de-identified and publicly accessible.

Single-cell count matrices were processed in R using Seurat (Satija et al., 2015; Stuart et al., 2019). Quality control was performed separately for each dataset using detected-gene number, total UMI count, and mitochondrial transcript proportion. After exclusion of low-quality cells, expression values were log-normalized, highly variable genes were selected, and the data were scaled. Principal-component analysis, graph-based clustering, and uniform manifold approximation and projection (UMAP) were then performed within each cohort.

### Refinement of epithelial compartments and singlet selection

Tumor-enriched epithelial objects were refined before state analysis by integrating cluster position, canonical marker expression, and contamination scores. Residual acinar-like populations were identified using PRSS1, PNLIP, and an acinar module score. In GSE212966, immune, stromal, and endothelial contamination scores were additionally evaluated. Clusters with concordant non-epithelial features were removed, and the retained epithelial cells were reclustered.

Potential doublets were identified separately within each biological sample or library using scDblFinder version 1.8.0 and raw RNA counts. Classifications were transferred to the cleaned epithelial objects by exact barcode matching. Cells classified as singlets constituted the principal population for subsequent state-level analyses.

### Definition of the composite epithelial state

The interferon-associated module (IFNcore) comprised STAT1, IRF1, ISG15, MX1, and IFI27. The epithelial-secretory module (Secretorycore) comprised S100P, PIGR, TFF1, AGR2, ELF3, CLDN3, and MUC13. These compact modules were selected to represent detectable interferon-response and ductal secretory programs within the epithelial compartments.

Module scores were calculated from the log-normalized RNA assay using Seurat AddModuleScore with its matched control-gene procedure. IFNcore1 and Secretorycore1 were standardized within each cohort, and the composite score was defined as VM_core = z (IFNcore1) + z (Secretorycore1). Cells at or above the cohort-specific 75th percentile were classified as VM-high, cells at or below the 25th percentile as VM-low, and the remaining cells as VM-intermediate. Continuous VM_core values were used for association analyses, whereas the three groups were used for visualization and high-versus-low comparisons.

### Molecular and sample-level characterization

Representative interferon-associated and epithelial-secretory genes were summarized in VM-high and VM-low cells within each cohort. Mean expression values were transformed to row-wise z scores for heatmap visualization. Differential expression between VM-high and VM-low cells was assessed within each dataset, and candidate genes were prioritized by considering both the expression contrast and the Spearman association between gene expression and continuous VM_core.

Biological consistency was evaluated at the sample level. IFNcore-Secretorycore Spearman correlations were calculated separately within each tumor. For IRF1, STAT1, and ELF3, VM-high versus VM-low contrasts were quantified within each sample using rank-biserial correlation, mean and median differences, and Hedges’ g. These sample-level summaries were used to assess direction and effect-size consistency across tumors and cohorts.

### Leave-one-gene-out and robustness analyses

Because IRF1 and STAT1 are included in IFNcore and ELF3 is included in Secretorycore, each candidate was removed in turn from its parent module. The reduced module and corresponding composite score were recalculated, and candidate expression was correlated with the reconstructed leave-one-gene-out VM score. This analysis assessed whether the associations persisted after removal of direct mathematical inclusion of the tested gene.

For cross-cohort robustness assessment, singlet epithelial objects were merged and embedded using Harmony version 1.2.3, with cohort-qualified sample or library identity as the batch variable. Harmony coordinates were used for neighborhood construction and visualization, whereas all biological scores and gene associations were calculated from the original normalized RNA assay. Large-scale copy-number patterns were inferred separately by sample using CopyKAT version 1.2.5. CopyKAT classifications were used only for sensitivity analyses of CNV-supported epithelial subsets and did not define the primary study population.

### External bulk validation and TCGA-PAAD analyses

GSE62452 included 69 PDAC tumors and 61 adjacent non-tumor tissues profiled using the Affymetrix Human Gene 1.0 ST platform. The normalized series matrix and GPL6244 annotation were used, and uniquely annotated probes were collapsed to gene-level values by the within-sample median. Bulk IFNcore, Secretorycore, and composite scores were calculated as arithmetic means of the detected genes. A non-overlapping interferon panel was used to test whether the composite score retained an interferon-associated context in an independent cohort.

In TCGA-PAAD primary tumors, the composite score was compared with a non-overlapping interferon validation score, curated immune, T-cell, and chemokine signatures, and ESTIMATE ImmuneScore, StromalScore, and TumorPurity (Yoshihara et al., 2013). Associations were assessed using Spearman correlation. For overall-survival analysis, GDC identifiers were mapped to primary-tumor cases, and survival time was defined from vital status, days to death, and the longest available follow-up. Kaplan-Meier curves used a median score split for visualization. Cox models analyzed the continuous standardized score; the principal multivariable model adjusted for age, sex, pathological stage, and histological grade.

### Statistical analysis

All statistical tests were two-sided. Wilcoxon rank-sum tests were used for two-group cell-level comparisons, and Spearman coefficients were used for continuous associations. Benjamini–Hochberg adjustment was applied where multiple comparisons were evaluated. Rank-biserial correlation and Hedges’ g were used to summarize within-sample effect sizes. Survival comparisons used the log-rank test and Cox proportional-hazards models, with hazard ratios reported per standard-deviation increase in the composite score. Cell-level tests were interpreted descriptively, while cross-sample direction and effect-size consistency were used to support biological reproducibility. A P value below 0.05 was considered statistically significant.

## Results

### Refined epithelial populations support state-level analysis

**FIGURE 1:**
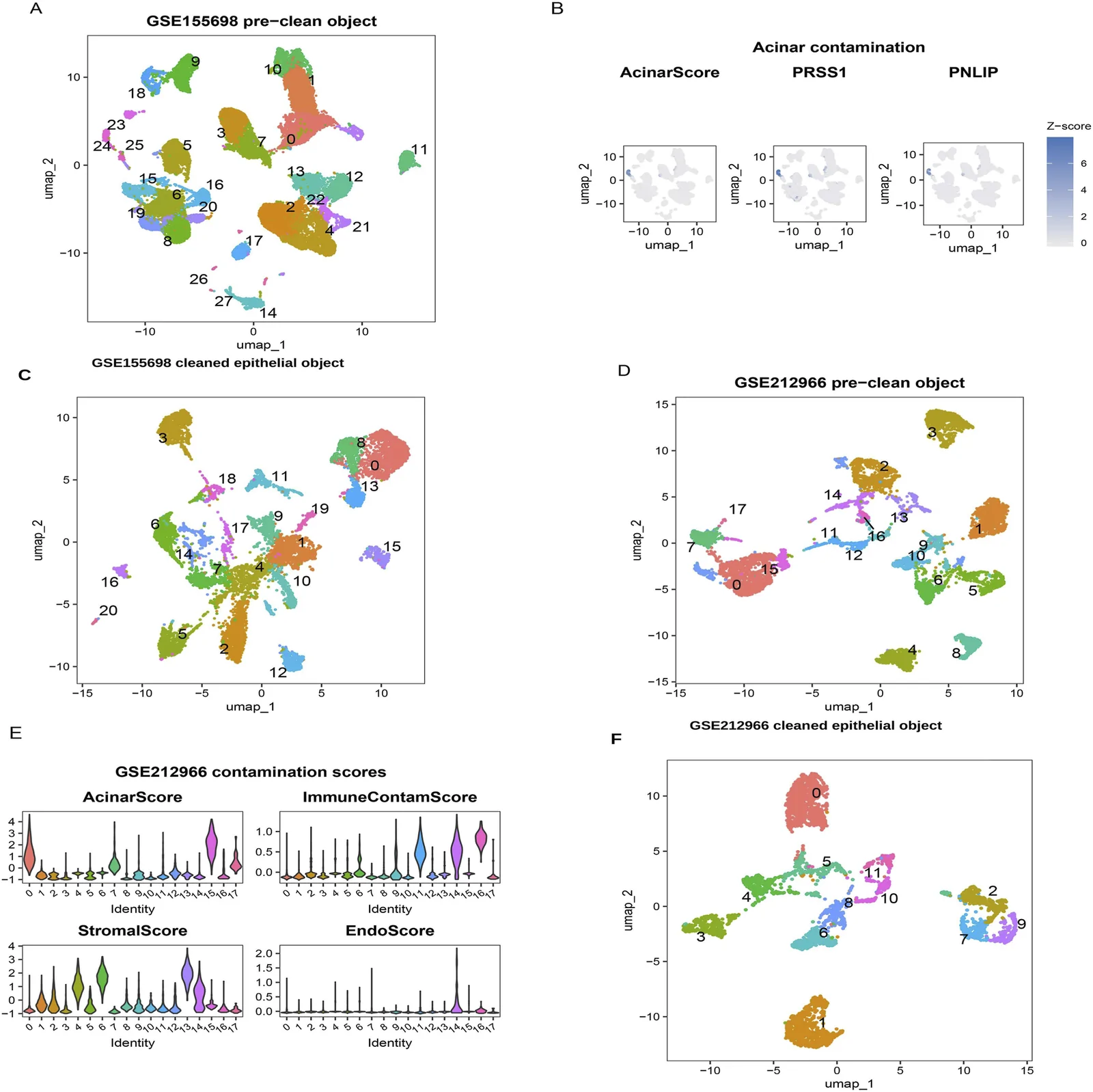
Quality-controlled definition of epithelial analysis compartments in two PDAC cohorts. **(A)** Uniform manifold approximation and projection (UMAP) of the GSE155698 tumor-enriched epithelial object before additional refinement. **(B)** AcinarScore and expression of PRSS1 and PNLIP identify residual acinar-like populations. **(C)** UMAP of the retained GSE155698 epithelial compartment after contaminating clusters were excluded and the cells were reclustered. **(D)** UMAP of the GSE212966 tumor-enriched epithelial object before additional refinement. **(E)** Cluster-level acinar, immune, stromal, and endothelial contamination scores in GSE212966. **(F)** UMAP of the retained GSE212966 epithelial compartment. Subsequent state-level analyses were restricted to cells classified as singlets by scDblFinder. Generated using the ggplot2 R package (Wickham, 2016).

Removal of residual acinar-like and other non-epithelial populations produced cleaned epithelial objects containing 10,237 cells in GSE155698 and 4,053 cells in GSE212966 (Figure 1). The refined objects retained broad epithelial heterogeneity while eliminating clusters with strong acinar, immune, stromal, or endothelial features. Sample-wise doublet assessment subsequently classified 9,812 GSE155698 cells and 3,875 GSE212966 cells as singlets, corresponding to more than 95% of each cleaned epithelial population (Supplementary Figure S2).

CopyKAT identified epithelial subsets with inferred large-scale copy-number alterations in both cohorts (Supplementary Figure S1). Candidate-gene associations remained generally directionally consistent in CNV-supported sensitivity analyses, although the classifications were heterogeneous. These findings supported the use of the cleaned singlet epithelial compartments for transcriptional-state analysis, with inferred CNV status treated as complementary rather than definitive evidence of malignant identity.

### A composite epithelial state integrates interferon-associated and secretory activity

**FIGURE 2:**
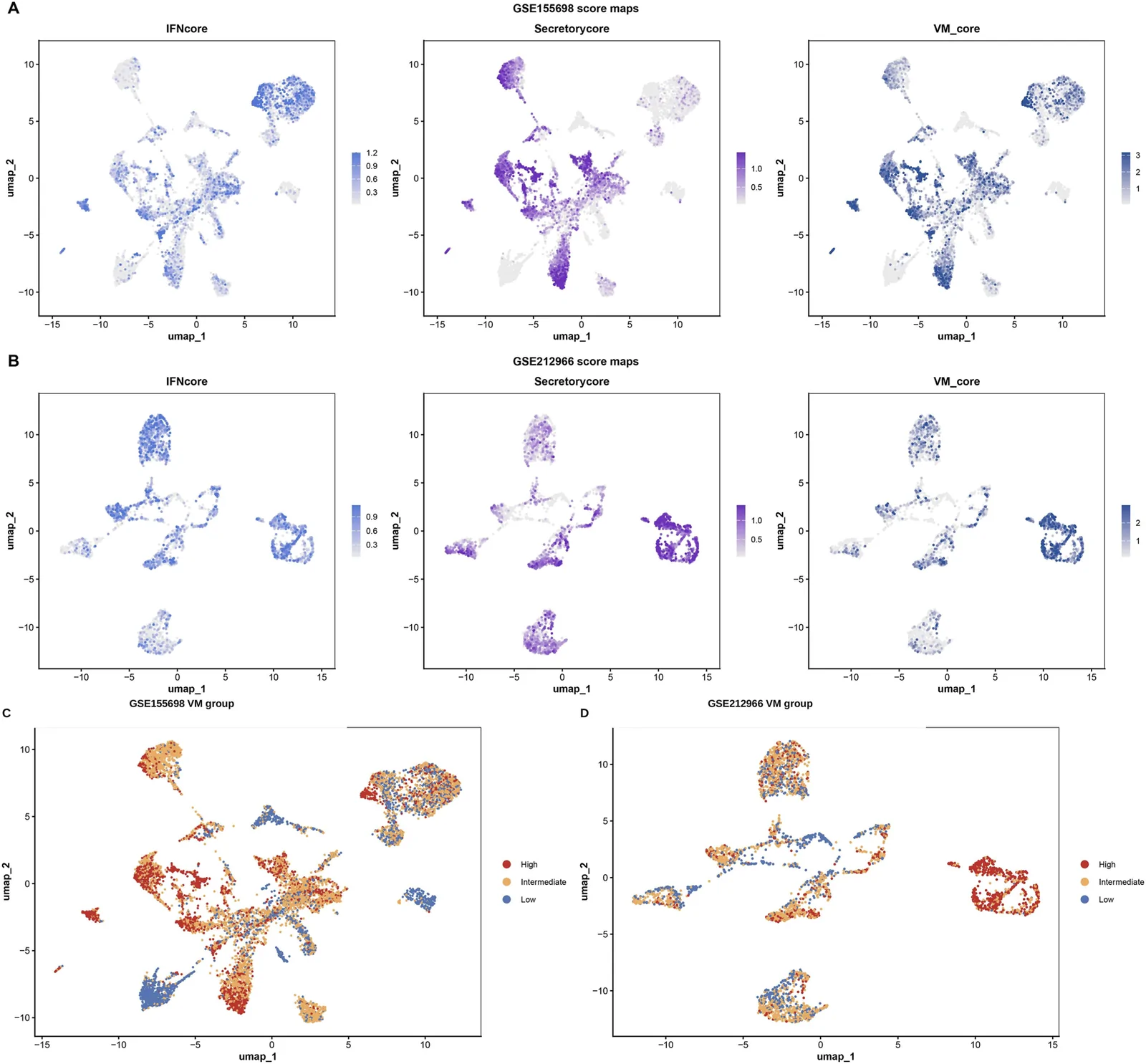
Single-cell distribution of interferon-associated and secretory activity within the composite epithelial state. **(A,B)** UMAPs show IFNcore, Secretorycore, and VM_core in cleaned epithelial cells from GSE155698 and GSE212966, respectively. **(C,D)** VM-high, VM-intermediate, and VM-low cells were defined independently in each cohort using the 75th and 25th percentiles of VM_core. Scores were calculated from log-normalized RNA expression and standardized within cohort. Generated using the ggplot2 R package (Wickham, 2016).

IFNcore and Secretorycore displayed structured but non-identical distributions across the epithelial landscapes of both cohorts. Their standardized combination, VM_core, identified cells with concurrent elevation of the two programs and separated VM-high, VM-intermediate, and VM-low populations using cohort-specific quartiles (Figure 2). VM-high cells were distributed across several epithelial subclusters rather than forming a single isolated cluster, indicating that the composite phenotype represented a continuous state superimposed on broader epithelial heterogeneity.

The same spatially distributed VM_core gradient remained visible after the two singlet datasets were represented in a Harmony-corrected coordinate system (Supplementary Figure S3). Thus, the state did not depend on a single cohort-specific embedding or on an isolated cluster boundary. The independent cohort analyses remained the basis for biological comparison, while the integrated representation provided complementary evidence that the combined interferon-associated and secretory pattern was preserved after batch-aware dimensional correction.

### VM-high epithelial cells share a conserved molecular program across cohorts

**FIGURE 3:**
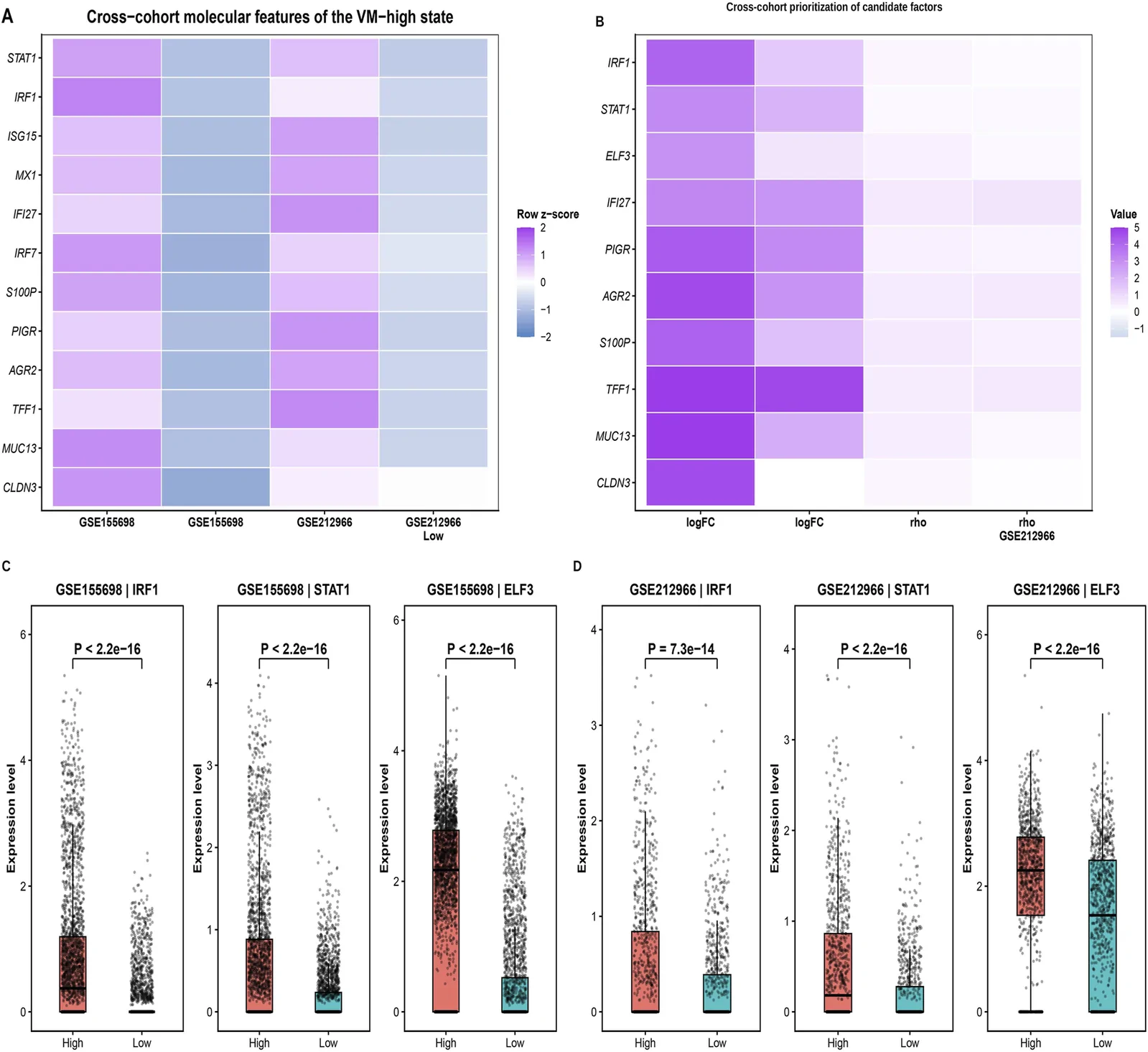
Conserved molecular features of VM-high epithelial cells across cohorts. **(A)** Cross-cohort heatmap of representative interferon-associated and epithelial-secretory genes in VM-high and VM-low cells. Values represent row-wise z-scored mean expression. **(B)** Candidate prioritization integrating VM-high versus VM-low differential expression and continuous Spearman association with VM_core. **(C,D)** Expression of IRF1, STAT1, and ELF3 in VM-high and VM-low cells from GSE155698 and GSE212966. In boxplots, center lines indicate medians, boxes indicate interquartile ranges, and whiskers extend to 1.5 times the interquartile range; points represent individual cells. Two-sided Wilcoxon rank-sum P values are shown. These comparisons identify candidate factors associated with the state and do not establish functional roles. Generated using the ggplot2 R package (Wickham, 2016).

VM-high cells in both cohorts showed coordinated enrichment of interferon-associated genes, including STAT1, IRF1, ISG15, MX1, IFI27, and IRF7, together with secretory epithelial genes such as S100P, PIGR, AGR2, TFF1, MUC13, and CLDN3 (Figure 3A). In contrast, both transcriptional components were attenuated in VM-low cells. The cross-cohort heatmap therefore revealed a conserved molecular configuration rather than enrichment of a single dominant module.

Combining VM-high versus VM-low expression changes with continuous gene-VM_core associations prioritized IRF1, STAT1, and ELF3 for focused evaluation (Figure 3B). All three genes were more highly expressed in VM-high cells in GSE155698 and GSE212966 (Figures 3C,D). IRF1 and STAT1 provided biologically interpretable anchors for the interferon-associated component, whereas ELF3 linked the composite state to epithelial-secretory identity. These results nominated the three genes as state-associated candidate factors without assigning causal or regulatory function.

### IRF1/STAT1 and ELF3 track distinct components of the composite state

**FIGURE 4:**
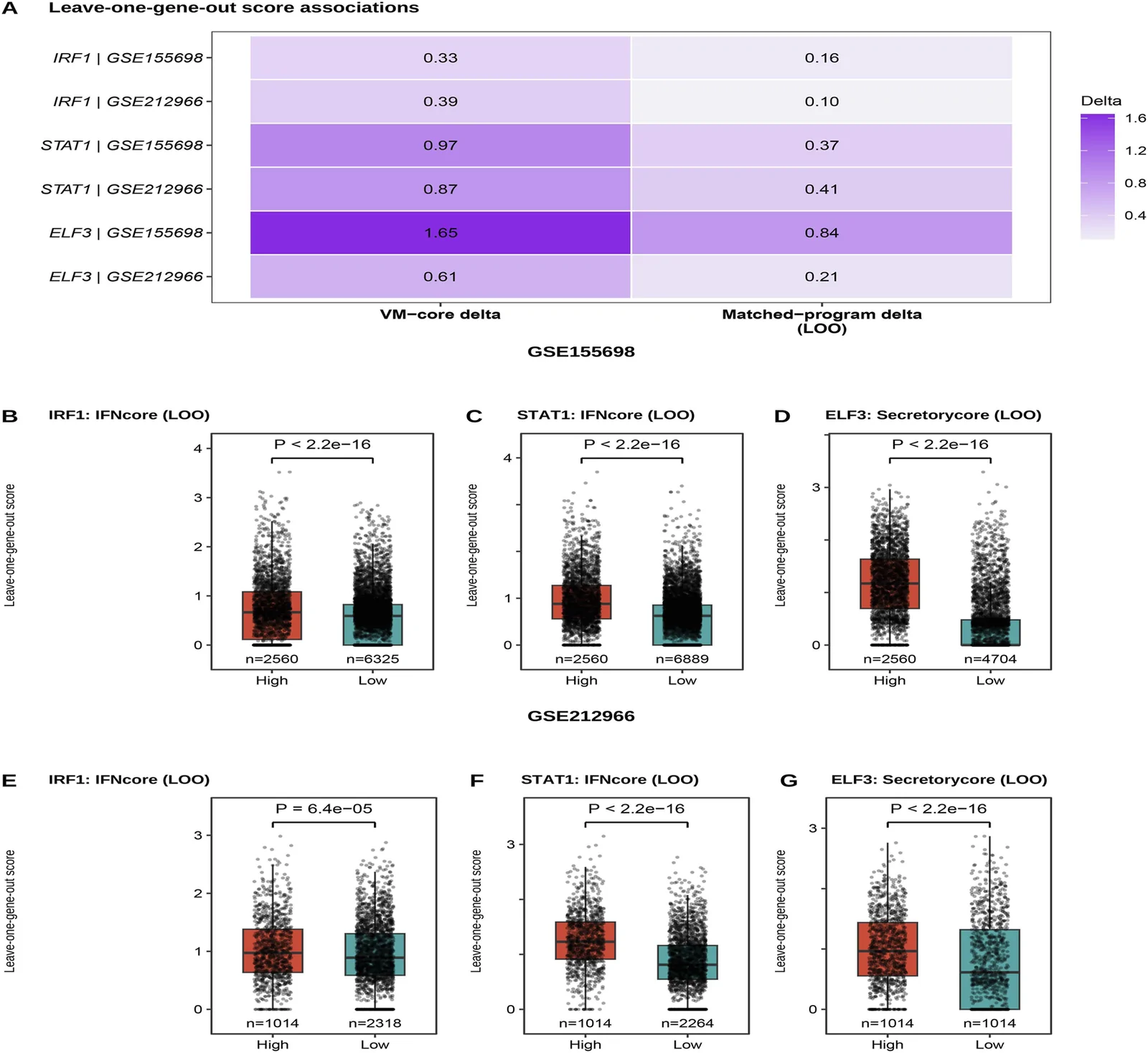
Candidate-factor associations after removal of direct score-gene overlap. **(A)** Summary of VM_core and matched-program score differences after IRF1, STAT1, or ELF3 was removed from its parent module. **(B–D)** GSE155698 comparisons of IRF1-associated IFNcore (LOO), STAT1-associated IFNcore (LOO), and ELF3-associated Secretorycore (LOO), respectively. **(E–G)** Corresponding comparisons in GSE212966. Center lines indicate medians, boxes indicate interquartile ranges, whiskers extend to 1.5 times the interquartile range, and points represent individual cells. Two-sided Wilcoxon rank-sum P values are shown. LOO, leave-one-gene-out. Generated using the ggplot2 R package (Wickham, 2016).

Leave-one-gene-out reconstruction showed that the candidate associations were not explained solely by direct inclusion of the tested genes in the original modules. After removal of IRF1 or STAT1 from IFNcore, cells with higher expression of the corresponding candidate retained higher reconstructed interferon and composite scores. Similarly, after removal of ELF3 from Secretorycore, ELF3-high cells retained higher reconstructed secretory and composite scores in both cohorts (Figure 4).

The continuous associations were also reproducible across individual tumors. Median within-sample correlations between candidate expression and the corresponding reconstructed VM score were 0.138 and 0.221 for IRF1, 0.271 and 0.317 for STAT1, and 0.457 and 0.134 for ELF3 in GSE155698 and GSE212966, respectively. VM-high versus VM-low effect sizes were positive in all evaluable tumors for IRF1 and STAT1 and in all but two GSE155698 tumors for ELF3 (Figure 6B). Together, these results support preferential alignment of IRF1/STAT1 with the interferon-associated component and ELF3 with the secretory component of the state.

### Bulk PDAC data support an interferon-linked epithelial context

**FIGURE 5:**
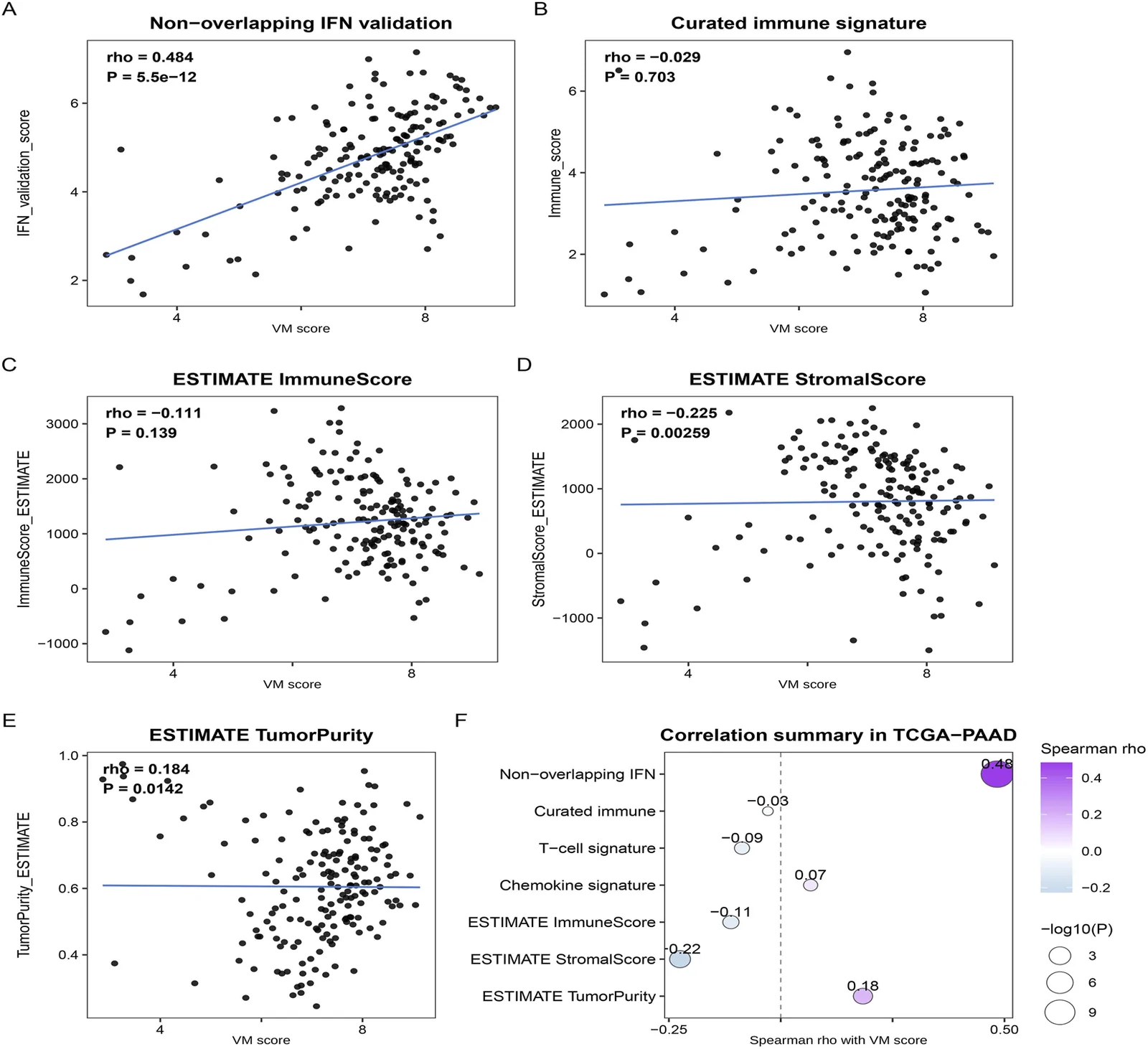
Bulk transcriptomic context of the composite signature in TCGA-PAAD. **(A)** Association between VM score and a non-overlapping interferon validation score. **(B)** Association with a curated immune signature. **(C–E)** Associations with ESTIMATE ImmuneScore, StromalScore, and TumorPurity. **(F)** Summary of Spearman associations with interferon-, immune-, stromal-, and purity-related metrics. Each point in panels A–E represents one TCGA-PAAD primary tumor; lines show linear fits for visualization, whereas reported coefficients and two-sided P values are from Spearman correlation. In panel F, point position and color indicate Spearman rho and point size indicates -log10(P). Generated using the ggplot2 R package (Wickham, 2016).

In TCGA-PAAD, the bulk composite score correlated with a non-overlapping interferon validation score (Spearman rho = 0.484, P = 5.5 × 10^−12^; Figure 5A). In contrast, associations with the curated immune signature and ESTIMATE ImmuneScore were weak and not statistically significant (Figures 5B,C). Correlations with StromalScore and TumorPurity were modest in magnitude (Figures 5D,E), and the summary analysis identified the independent interferon program as the dominant tissue-level correlate of the composite signature (Figure 5F).

These results indicate that the bulk signature was not readily explained by broad immune-cell abundance or stromal enrichment. However, because bulk profiles average signals across malignant, stromal, and immune compartments, they cannot establish the cellular source of the association. The TCGA analysis therefore provides tissue-level context for the single-cell observation rather than direct confirmation of cell-intrinsic co-expression.

### Patient-level and external validation establish reproducibility across cohorts

**FIGURE 6:**
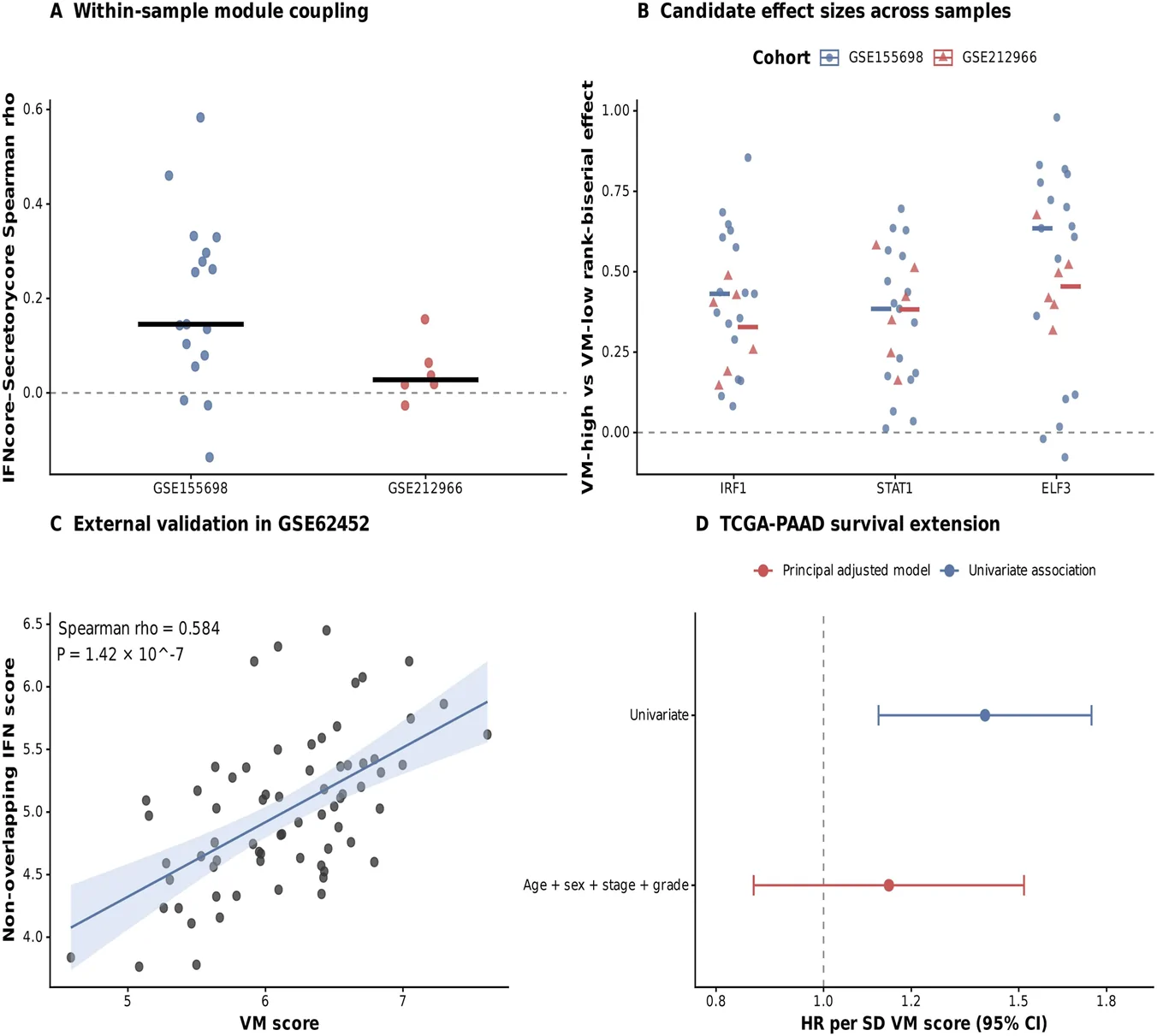
Patient-level, external, and clinical support for the composite epithelial state. **(A)** Within-tumor Spearman correlations between IFNcore and Secretorycore in the two single-cell cohorts. Each point represents one PDAC tumor and black bars indicate cohort medians. **(B)** Sample-level rank-biserial effects comparing VM-high with VM-low expression of IRF1, STAT1, and ELF3; each point represents one tumor and colored bars indicate cohort medians. Positive values indicate higher expression in VM-high cells. **(C)** Association between VM score and the non-overlapping interferon score among 69 GSE62452 PDAC tumors; the line and shaded band show the linear fit and 95% confidence interval for visualization, and the reported statistic is Spearman rho. **(D)** Hazard ratios (HRs) and 95% confidence intervals (CIs) per one-standard-deviation increase in continuous VM score in TCGA-PAAD. The principal multivariable model adjusted for age, sex, stage, and grade. Generated using the ggplot2 R package (Wickham, 2016).

Within individual PDAC tumors, IFNcore and Secretorycore were positively associated in 14 of 17 GSE155698 samples and 5 of 6 GSE212966 samples (Figure 6A). Median within-sample correlations were 0.145 and 0.028, respectively. Although coupling strength differed between cohorts, the positive direction was reproduced in more than 80% of evaluable tumors in each dataset, providing patient-level support beyond pooled-cell comparisons.

Independent validation in 69 GSE62452 tumors showed that the composite score correlated with a non-overlapping interferon score (rho = 0.584, P = 1.42 × 10^−7^) and that IFNcore correlated with Secretorycore (rho = 0.276, P = 0.0219; Figure 6C; Supplementary Figure S4). In TCGA-PAAD, a higher continuous composite score was associated with poorer overall survival in univariate analysis (HR per standard deviation = 1.40, 95% CI 1.12–1.75; P = 0.0030), but the association was attenuated after adjustment for age, sex, stage, and grade (HR = 1.15, 95% CI 0.87–1.52; P = 0.343; Figure 6D; Supplementary Figure S5). Thus, the state showed reproducible biological and external transcriptomic support, while independent prognostic value was not established.

## Discussion

This study identifies a reproducible PDAC epithelial transcriptional state in which interferon-associated activity coexists with a ductal secretory program. The state was observed independently in two single-cell cohorts, showed positive module co-variation in more than 80% of evaluable tumors in each cohort, and was supported by consistent sample-level candidate effects and external bulk validation. These findings extend previous descriptions of PDAC epithelial heterogeneity by resolving a recurrent state that is not apparent from conventional subtype labels alone (Peng et al., 2019; Lin et al., 2020; Elyada et al., 2019; Wang et al., 2021; Cui Zhou et al., 2022; Werba et al., 2023; Oh et al., 2023; Loveless et al., 2025).

The biological feature that distinguishes this state is the maintenance of secretory epithelial identity in cells with increased interferon-associated activity. Interferon signaling in PDAC can reflect inflammatory pressure, innate immune sensing, treatment exposure, or cell-intrinsic stress adaptation (Muthalagu et al., 2020; Ischenko et al., 2021; Vonderhaar et al., 2021; Perevalova et al., 2024; Alsamman and El-Masry, 2018). Its coexistence with S100P-, PIGR-, AGR2-, TFF1-, MUC13-, and CLDN3-associated expression suggests that an inflammatory response can be superimposed on a differentiated ductal-secretory program rather than replacing epithelial identity.

The distribution of VM_core across multiple epithelial subclusters further argues that the phenotype is better viewed as a continuous transcriptional axis than as a discrete lineage. Such an axis could intersect established classical and basal-like states, providing an additional dimension of epithelial plasticity. Determining its precise relationship to existing PDAC subtype frameworks will require datasets in which subtype, treatment history, and spatial context can be evaluated simultaneously.

IRF1, STAT1, and ELF3 provide biologically interpretable anchors for this state. IRF1 and STAT1 are central components of interferon-response signaling (Muthalagu et al., 2020; Ischenko et al., 2021; Vonderhaar et al., 2021; Perevalova et al., 2024; Alsamman and El-Masry, 2018), whereas ELF3 is linked to epithelial differentiation and maintenance of epithelial transcriptional programs (Subbalakshmi et al., 2023; Luk et al., 2018). Their consistent sample-level effects and persistence after leave-one-gene-out score reconstruction support association with different components of the state. However, these analyses cannot determine whether the candidates initiate, maintain, or merely mark the phenotype. Perturbation studies that measure both transcriptional arms and relevant cellular phenotypes will be required to establish causality.

The external analyses provide complementary context. GSE62452 reproduced the association between the composite score and an independent interferon signature, supporting generalizability beyond the discovery cohorts. In TCGA-PAAD, the univariate association with overall survival suggests that the state may capture clinically relevant disease biology, although the attenuation after clinicopathological adjustment indicates overlap with established disease features rather than independent prognostic performance. The signature should therefore be considered a biological state descriptor, not a validated prognostic biomarker.

Several limitations should be acknowledged. The study is based on public transcriptomic datasets, and bulk validation cannot establish that both programs are co-expressed within the same cells. CopyKAT provides computational support for CNV-altered epithelial subsets but cannot establish the identity of every cell. Harmony and doublet analyses reduce specific technical concerns but cannot remove all cohort and sampling differences. Most importantly, observational transcriptomics does not establish function. Future cell-resolved and spatial studies, followed by experimental perturbation, should determine the tissue localization, stability, and phenotypic consequences of this epithelial state.

## Data Availability

The original contributions presented in the study are publicly available. This data can be found in the GEO repository with the accession numbers GSE155698 and GSE212966.
